# Computing Joint Action Costs: Co-Actors Minimize the Aggregate Individual Costs in an Action Sequence

**DOI:** 10.1162/opmi_a_00045

**Published:** 2021-09-10

**Authors:** Georgina Török, Oana Stanciu, Natalie Sebanz, Gergely Csibra

**Affiliations:** Department of Cognitive Science, Central European University; Department of Cognitive Science, Central European University; Department of Cognitive Science, Central European University; Department of Cognitive Science, Central European University; Department of Psychological Sciences, Birkbeck, University of London

**Keywords:** joint action, cost computation, efficiency, decision making, cooperation

## Abstract

Successful performance in cooperative activities relies on efficient task distribution between co-actors. Previous research found that people often forgo individual efficiency in favor of co-efficiency (i.e., joint-cost minimization) when planning a joint action. The present study investigated the cost computations underlying co-efficient decisions. We report a series of experiments that tested the hypothesis that people compute the joint costs of a cooperative action sequence by summing the individual action costs of their co-actor and themselves. We independently manipulated the parameters quantifying individual and joint action costs and tested their effects on decision making by fitting and comparing Bayesian logistic regression models. Our hypothesis was confirmed: people weighed their own and their partner’s costs similarly to estimate the joint action costs as the sum of the two individual parameters. Participants minimized the aggregate cost to ensure co-efficiency. The results provide empirical support for behavioral economics and computational approaches that formalize cooperation as joint utility maximization based on a weighted sum of individual action costs.

## INTRODUCTION

Humans cooperate by sharing goals with others, and by planning and coordinating actions with their partners to achieve these goals (Bratman, 1992; Butterfill, 2017; Sebanz et al., 2006). Everyday social interactions, such as assembling furniture with a friend, or cooking a meal together, attest to the complexity of planning required in cooperative activities. For instance, family members might share the overarching goal of cooking a paella: each of them represents and works toward specific subgoals (e.g., chopping vegetables), and they need to distribute the necessary actions among themselves. Many subtasks contribute to the joint goal of cooking a paella. Accordingly, these actions may be executed in many different ways, with varying degrees of efficiency. When people distribute subtasks between themselves, the individual efficiencies of co-actors often depend on each other; in some situations, they are inversely related. That is, because a complex joint action may be composed of many interdependent subtasks, performing a less effortful subtask may force the other person to contribute a more effortful complementary action to ensure the success of the joint action.

Planning cooperative sequences can be regarded as making a series of decisions about actions to be performed by co-actors (cf. Wolpert & Landy, 2012, on individual motor planning). What principles guide people’s decision making? Previous studies suggest that co-actors tend to maximize the joint efficiency of an action sequence by minimizing the total costs of movements when they work toward a shared goal (Kleiman-Weiner et al., 2016; Santamaria & Rosenbaum, 2011; Török et al., 2019). These findings are paralleled by evidence that in certain economic games, people sometimes make decisions consistent with a collective utility-maximizing strategy based on team reasoning (Sugden, 2003), rather than choosing individual utility-maximizing solutions (Colman et al., 2008). We argue that such behaviors do not only appear in contexts with financial rewards at stake.

In the joint action task of Török and colleagues (2019) participants made binary decisions between two action plans to coordinate their hand movements with a partner in a sequential manner. One of the two options was more efficient for the decision-making actor (i.e., the initiator of the action sequence), the other option was more efficient for her partner, therefore the jointly efficient plan was more individually efficient for either the decision maker or for the partner. The participants tended to make *co-efficient* (i.e., jointly efficient) rather than individually efficient decisions that would have either maximized personal efficiency or would have altruistically increased the utility of a partner (cf. Axelrod & Hamilton, 1981; Trivers, 1971). The present study investigated the computations behind decisions that minimize the aggregate costs of the group.

To minimize a dyad’s costs in action planning, a decision maker first needs to estimate them (Körding & Wolpert, 2006). In the case of joint actions, we assume that the expected individual costs of potential joint action sequences are integrated to achieve optimal decisions. People are sensitive to their real or virtual partner’s individual efforts, needs, and task difficulty (Chennells & Michael, 2018; Ray et al., 2017; Ray & Welsh, 2011). We hypothesize that, whenever this is calculable, the costs of joint actions are estimated as the summed total of individual costs. This proposal is compatible with computational work in which joint utility is represented as the weighted sum of the individual utilities of each agent (Kleiman-Weiner et al., 2016).

While assessing and summing individual costs may be a generic process to achieve co-efficiency (applicable when we conceptualize the joint cost estimation problem in the abstract, akin to a mathematical problem of combining two quantities), depending on the actual context, shortcuts may also be available. For example, the task in our study (Török et al., 2019) required two actors to move an object along one of two paths by taking turns. While the movement was divided between the participants, the decision-making actor could have planned the joint action sequence as if she had intended to complete the task alone, and then performed only the first section of the plan. Such a planning process would result in choosing the co-efficient action option from the alternatives without requiring the planner to sum individual costs.

In the present study, we employed a task in which joint action costs cannot be computed without representing and summing two individual action costs. The participants had to move objects on a touchscreen sequentially,^1^ and the cost of this action was assumed to be proportional to the path length of movement. Crucially, the physical separation of paths to be taken by co-actors made it impossible to plan a single action that incorporated both paths. This feature of the task enabled us to generate, and parametrically vary, individual and joint action costs in various ways. Since a priori we could not exclude the possibility that participants might adapt to the correlational structure of the cost parameters during the experiment, we used separate participant samples for three versions of the task in which pairs of cost parameters were decorrelated. We observed highly consistent results across experiments, and thus we report the analyses of pooled data. The individual experiments’ details are available in the Supplemental Materials (section S3 and Table S3.1).

If people represent the joint costs of an action sequence as a weighted sum of individual costs, choices between action plans should be consistent with a co-efficiency-maximizing strategy that minimizes this sum. We hypothesized that, in the absence of asymmetries in social hierarchy (Kleiman-Weiner et al., 2016), the individual costs of the actors would be weighed equally in the sum.

We also investigated an alternative hypothesis, according to which the equality of contribution matters more than the efficiency of joint performance. People are often motivated to reduce payoff inequality in economic games (Dawes et al., 2007; Fehr & Schmidt, 1999), and it is possible that in a joint action context they minimize the difference in the action costs distributed across co-actors rather than maximizing the expected utility of the dyad. Such decisions may be based on a motivation to be fair to an interaction partner (Rand et al., 2013), although recent results support the co-efficiency hypothesis against the trial-based fairness account (Strachan & Török, 2020).

## METHODS

### Participants

We recruited participants through Central European University’s Research Participation System (SONA Systems) and a student job agency. They gave their informed consent and received vouchers for their participation. The study was approved by the United Ethical Review Committee for Research in Psychology (EPKEB) in Hungary. To ensure that the present study was adequately powered to make inferences in the Bayesian model, the target sample size was set to 20 dyads (40 participants) per experiment, a larger sample size relative to our previous study (Török et al., 2019). We present the data of 120 participants (82 females, 2 preferred not to specify; *M*_age_ = 23.81 years, *SD* = 4.07) (see section S1.1 for descriptions of exclusions).

### Apparatus

The task was performed on a touchscreen monitor (Iiyama PROLITE 46″, resolution 1920 × 1080 pixels, separate sync-horizontal: 31.47–67.5 KHz, vertical: 47–63 Hz) lying flat on a table between two participants facing each other. Stimulus presentation and data recording were controlled by a script using the Psychophysics Toolbox (Brainard, 1997; Kleiner et al., 2007; Pelli, 1997) in MATLAB®. Two response boxes (Black Box Toolkit Ltd.) were used to control trial onset.

### Stimuli and Task

**Figure 1.**
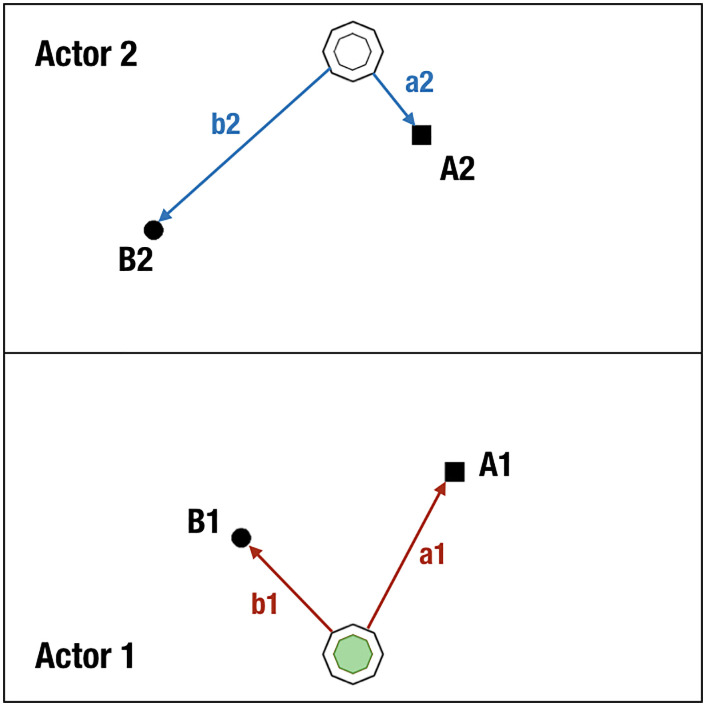
**An example of the layouts presented to the participants.** Starting locations were indicated by the octagons, and the locations of the two pairs of black target objects (circles and squares) were generated by stochastic selection processes. The arrows and labels (not shown to the participants) indicate the distances between the starting locations and the target objects, which provided the basis for cost calculations comparing the two target options.

On each trial, a layout displaying the following elements was presented to the participants: (1) a thin black wall dividing the screen into the two participants’ task areas, (2) two pairs of black target objects (two circles and two squares, 30 px diameter) distributed between the task areas, and (3) two black-bordered octagonal starting locations (96 × 96 px) with another, small octagon inside (60 × 60 px, Figure 1). The starting locations were always displayed at mid-position along the longer sides of the screen, aligned with the response box buttons.

Participants were instructed to keep their dominant index finger on the button of their response boxes to trigger the start of each trial. At the beginning of each trial, one of the smaller octagons was orange-colored to signal which participant would initiate the joint action as Actor 1. In each trial, Actor 1 had to choose between the two target objects on her side and drag it back to her starting location. The participants were instructed to inspect the layout while the octagon was orange-colored, and to decide which target object they would pick up when prompted to move.

After 3 s, the color switched to green, which served as a cue for Actor 1 to start moving. By dragging the green octagon over a black object with her index finger, the participant picked up the object and collected it by transferring it back to her starting location. Once Actor 1 collected one of the objects, she pressed the button on her response box again to make the white octagon in front of her partner (Actor 2) turn green. The appearance of this second green octagon cued Actor 2 to start moving to collect the matching object in his task area. The trial was over when Actor 2 collected the object with the shape corresponding to the one chosen by Actor 1 (nonmatching objects did not respond to dragging). Thus, while both participants acted in each trial, only Actor 1 made the decision that determined the individual and joint costs incurred during the completion of the task.

### Design

We considered the cost of an action as a monotonic function of the path length that the object covered on the touchscreen when dragged, and, for the sake of simplicity, we treated the path length as the absolute cost paid for its transport. For example, in Figure 1, the cost of choosing object A1 (the square) is the distance between Actor 1’s starting location and this square: a1. If Actor 1 makes her decision based on her expected cost, she should compare this cost to the cost of moving object B1: b1. The cost disparity between these actions is expressed by their difference: a1 − b1. We call this value Self Cost Disparity, or simply Self Disparity. If Actor 1 intends to make individually efficient decisions, she should choose A1 as the target when the Self Cost Disparity is negative, and B1 when this value is positive (as is the case in Figure 1). The matching individual cost disparity for Actor 2 (Other Disparity, a2 − b2) in this example is negative. Thus, picking up object B1 would be individually optimal for Actor 1 because it minimizes her Self Disparity, whereas it is the less efficient option for Actor 2.

The joint cost of an action is taken to be the summed costs of the actors. If Actor 1 chooses A1, the joint cost is a1 + a2; if she chooses B1, the joint cost is b1 + b2. Thus, the Joint Cost Disparity (or Joint Disparity) is (a1 + a2) − (b1 + b2), which is the sum of the two individual disparities (Self Disparity and Other Disparity). In the example arrangement (Figure 1), the Joint Disparity is negative, suggesting that from the dyad’s perspective, collecting the square objects was associated with the shorter total path length, and as such, was the co-efficient choice. At the same time, picking up the square object pair was also individually efficient for Actor 2 (negative Other Disparity), but not for Actor 1 (positive Self Disparity).

We assume that the likelihood of choosing object A1, which was always the square in the decision maker’s side of the screen, parametrically depends on the magnitude of one or more of these disparities through a logistic link function. For example, if Actor 1 optimizes her own cost, the more negative the value of Self Disparity, the more likely it is that she will choose A1, forcing Actor 2 to act on A2.

To generate the target objects’ locations, we first sampled Self Disparity and Other Disparity (section S1.2 reports details of the sampling procedure). The positions of the objects were then randomly selected to match these disparities. For each dyad, 100 different spatial arrangements were generated and repeated, once per each participant acting as Actor 1. Trial order was pseudorandomized, with the constraint that neither of the participants be assigned the role of Actor 1 more than three times in a row.

To address our alternative hypothesis, we operationalized Fairness as the difference between the asymmetries in individual paths related to object pair A and object pair B distributed between co-actors in each trial: the difference between [abs(a1 − a2)] and [abs(b1 − b2)] (Figure 1). Choices were considered “fair” if the object Actor 1 picked up was associated with a relatively smaller difference in the path lengths between Actors 1 and 2 than the path length difference associated with the alternative object. When the fairness measure was negative, choosing object A1 was the fair option; when positive, object B1 was fair.

### Procedure

Before the object matching task, the participants read step-by-step instructions on how to complete a trial. They were instructed to collect matching object pairs by cooperating with their partner, without communicating, and to complete each trial as quickly as possible. The participants first completed six practice trials to familiarize themselves with the task, the touchscreen, and the response box buttons. They then completed the main task (on average in *M* = 34.62 minutes, *SD* = 4.99) without receiving any feedback.

### Data Analysis

To test whether object choices were influenced primarily by the difference between joint action costs, rather than between the individual costs of Actor 1 or Actor 2, we fitted and contrasted three mixed-effects logistic regression models using a Bayesian estimation technique (Kruschke, 2015; details of the hierarchical model are reported in S1.3). Specifically, the probability of choosing object A1 was predicted, in turn, by (1) *Self Disparity*, (2) *Other Disparity*, and (3) a weighted linear combination of the *Self and Other Disparities*. We expected that the third model would have the best fit to the data, since it is the only one that can express the co-efficient strategy, which dictates that actors should equally weigh their own and their partner’s cost disparities. Additionally, we fitted models that predicted choices by (4) Fairness or by (5) the linear combination of Self and Other Disparities and Fairness.

The posterior distributions of the beta coefficients were estimated in JAGS (Plummer, 2003) with the runjags package in R (Denwood, 2016). We report the population-level estimates. To compare models, we calculated leave-one-out cross-validation measures (LOO-CV; Gelman et al., 2014; Vehtari et al., 2017) using the loo package in R (Vehtari et al., 2020). For each model, we report an estimation of the expected log predictive density converted to the deviance scale (i.e., multiplied by −2), so that it is comparable to other information criteria. The lower this leave-one-out information criterion (LOOIC) for a model, the better its expected accuracy at out-of-sample prediction of future data. We also compared area under the curve (AUC) measures for each model (Fawcett, 2006), quantifying model fit to the observed data. We base model comparison on the AUC and LOOIC (see Table S2.1).

## RESULTS

### Descriptive Statistics

The participants chose object A1 on 49.7% of trials. The individual object choice proportions were not different from chance (Wilcoxon signed-rank test against 0.5: *V* = 2724, *p* = .726, rank-biserial correlation *r* = −.25, 95% confidence interval (CI) for proportion .50 = [.49, .51]).

#### Cost-Minimization

**Figure 2.**
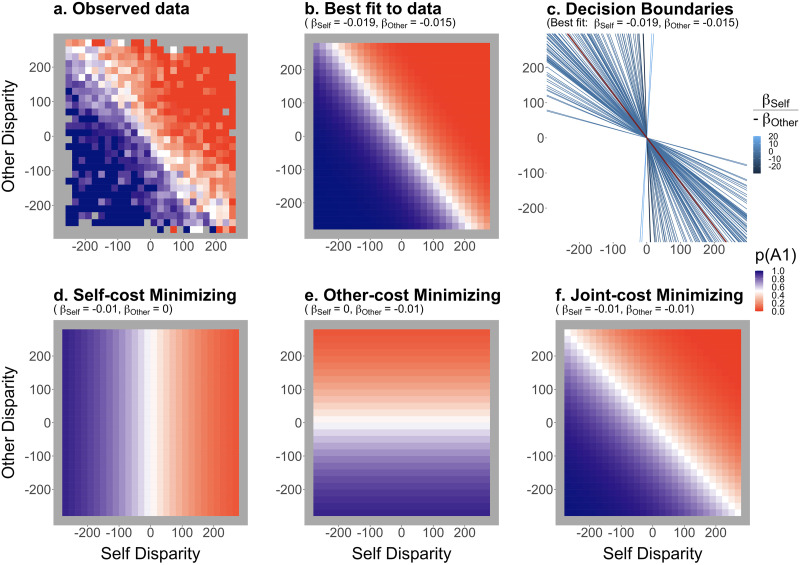
**Results of the experiments against the predictions of the main cost-minimization strategies.** (a) Observed object A1 choices, and (b) the posterior predictions of the best-fitting model using the linear combination of Self and Other Disparities. (c) Individual decision boundaries according to the best-fitting model. The red line indicates the population-level boundary. (d–f) Predictions for optimal responses according to Self-, Other-, and Joint-cost (i.e., Self + Other costs) minimizing strategies, respectively. The lower the disparity to be minimized according to a model, the higher the probability of picking object A1 (blue). Predictions were calculated assuming that one pixel increase in a given parameter would result in 1% decrease in the odds of choosing A1 over B1. All plots feature disparities in pixels.

The magnitudes of cost disparities strongly influenced object choices. Overall, participants chose the object resulting in a co-efficient action sequence 77% of the time (95% CI for proportion .77 = [.76, .79]), which was significantly higher than chance at 66%^2^ (*V* = 7260, *p* < .001, *r* = 1.00). The participants’ decisions are illustrated in Figure 2a, together with the predictions calculated for each cost-minimizing strategy (Figure 2d–f).

#### Fairness

**Figure 3.**
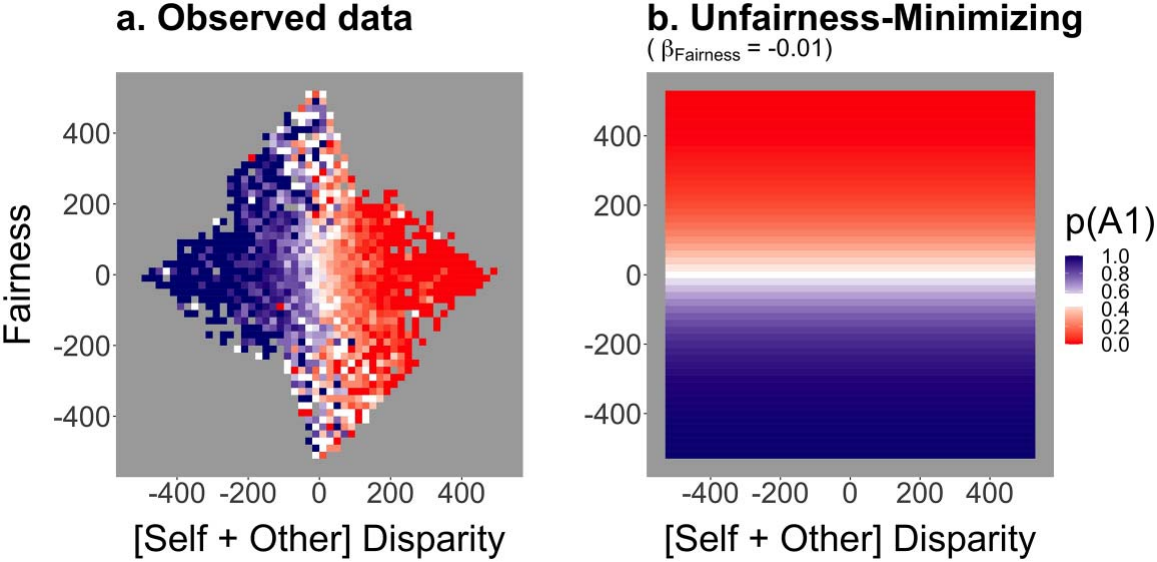
**Observed object choices against the predictions of an Unfairness-Minimizing strategy.** (a) Observed object A1 choices as a function of Fairness and the linear combination of Self and Other Disparity. (b) Predictions for optimal responses according to a strategy that minimizes the unfairness of task distribution between co-actors. The lower the degree of asymmetry in costs (or the magnitude of joint disparities) the higher the probability of picking object A1. The predictions were calculated assuming that one pixel increase in unfairness would result in 1% decrease in the odds of choosing A1 over B1. Both plots feature disparities in pixels.

The participants made fair choices 47.5% of the time (95% CI for proportion .48 = [.46, .50]), which was not significantly different from chance (*V* = 2479, *p* = .047^3^, *r* = −.32). Decisions were more strongly influenced by Joint-cost minimizing concerns than by Fairness (Figure 3).

### Parameter Estimations

The posterior modes and 95% highest density intervals (HDI) for the population-level parameters that represent how participants weighted the cost disparities to make object choices are presented in Table 1 for all of the models fitted. Experiment-level parameter estimates are summarized in the Supplemental Materials (section S2, Table S2.1).

**Table 1.** Parameter estimates from all fitted models, with measures of predictive accuracy and model fit (LOOIC—leave-one-out information criterion, AUC—area under the curve).

**Object choice (A1) predictors**	**Mode of posterior [95% HDI]**			**LOOIC [*SE*]**	**AUC**
	*μ*_βSelf_	*μ*_βOther_	*μ*_βFairness_		
(1) Self Disparity	−0.156 [−0.313, −0.005]			13109.1 [97.3]	0.735
(2) Other Disparity		−0.058 [−0.210, 0.092]		14529.2 [82.0]	0.628
(3) Self Disparity and Other Disparity	−0.367 [−0.469, −0.273]	−0.291 [−0.390, −0.193]		9437.8 [124.6]	0.859
(4) Fairness			−0.003 [−0.089, 0.092]	16153.9 [42.2]	0.542
(5) Self Disparity, Other Disparity, and Fairness	−0.376 [−0.475, −0.256]	−0.282 [−0.397, −0.185]	−0.031 [−0.120, 0.083]	9407.8 [124.5]	0.851
1. (3.2) Self Disparity and Other Disparity (unambiguous trials)	−0.348 [−0.440, −0.246]	−0.267 [−0.378, −0.146]		5414.6 [93.5]	0.855
2. (3.3) Self Disparity and Other Disparity (Block 1)	−0.298 [−0.414, −0.193]	−0.110 [−0.216, −4.08e−05]		610.6 [27.2]	0.825
3. (4.2) Fairness (unambiguous trials)			0.172 [0.038, 0.318]	7850.9 [75.0]	0.748

*Note*. Coefficients were rescaled to express the effect of the cost disparities in units of one on-screen cm. Pixel-based estimates for all parameters are reported in Table S2.1.

**Figure 4.**
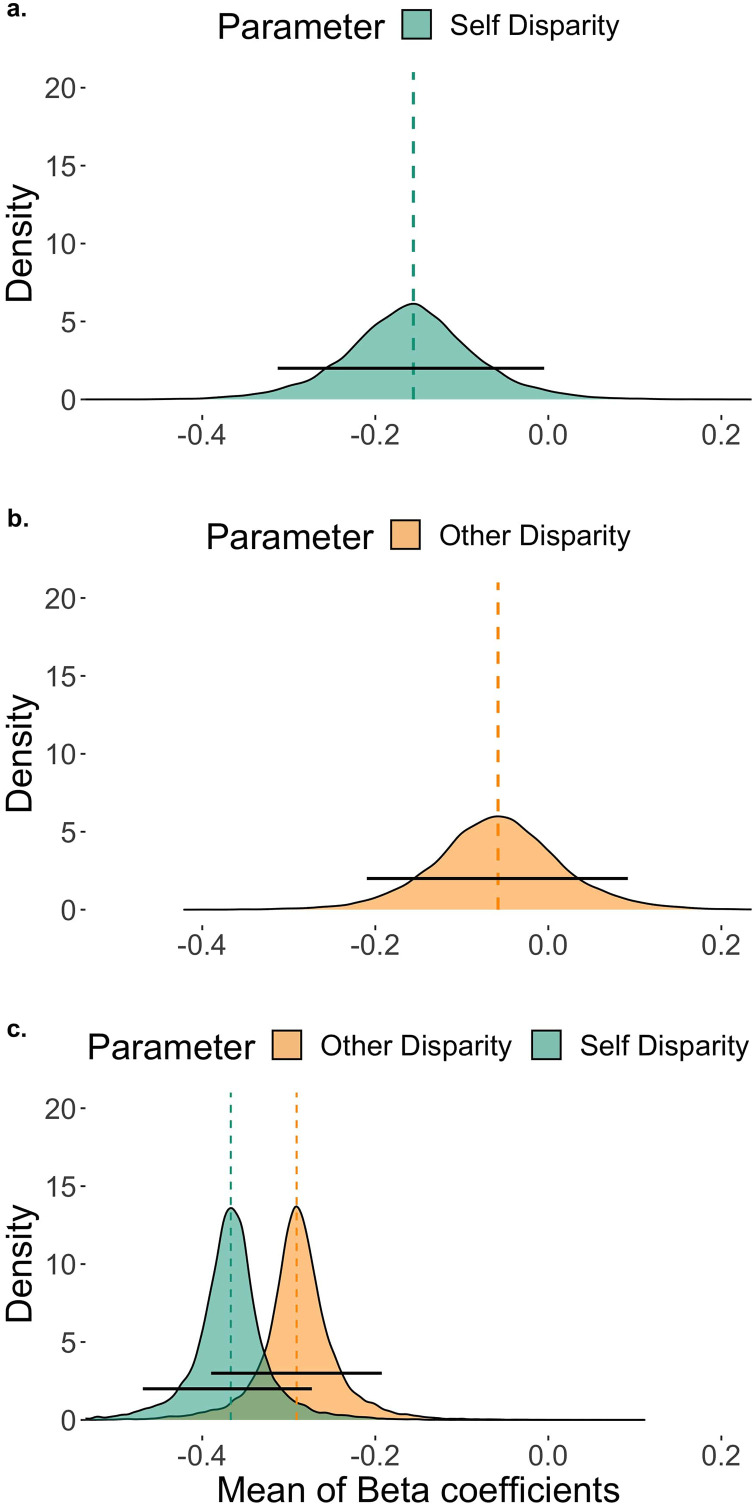
**Parameter estimates for the Self-, Other-, and Joint-cost minimization models.** (a)–(c) Posterior probability distributions of the rescaled *μ*_β_ parameters for Self and Other Disparities in Models 1 to 3 (see Table 1), respectively. The dashed vertical lines indicate the modes of *μ*_β_, the black horizontal lines represent the 95% HDIs (highest density intervals).

Turning first to the main hypothesis on the relative weighting of the Self and Other Disparities, a model including both disparities was a better fit for the data than models including either disparity alone. In this two-predictor model, both population-level means (*μ*_βSelf_ and *μ*_βOther_) of the coefficients for the disparities were distributed below zero (Figure 4c, Self: 95% HDI: [−0.469, −0.273], Mode *μ*_βSelf_ = −0.367; Other: 95% HDI: [−0.390, −0.193], Mode *μ*_βOther_ = −0.291). This suggests that both parameters made non-null contributions in the predicted directions. Increasing Self and Other disparities by a centimeter led to a 30.7% and 25.2% decrease in the odds of picking A1 over B1, respectively.

The 95% HDI of the posterior of the difference between the disparities’ coefficients included zero (Mode_diff_(*μ*_βSelf_ − *μ*_βOther_) = −0.072, 95% HDI = [−0.214, 0.052]). In a given trial, the likeliest expected decrease in the odds of an A1 over a B1 choice was 7% larger due to a one-centimeter increase in Self Disparity than due to a one-centimeter increase in Other Disparity. In other words, the likeliest estimated overall effect of Self Disparity on the probability of a square object choice was 7% larger than the effect of Other Disparity. The possible odds changes, however, ranged from a 19.2% larger relative decrease of the odds based on Self Disparity to a 5.4% larger relative decrease based on Other Disparity. These results suggest that the difference between the magnitudes of the two disparities’ effects on decision making was credibly small across the experiments. The average relative weights on Self and Other Disparity were .56 (95% HDI = [.42, .71]) and .44 (95% HDI = [.29, .59]), respectively (for participant-wise estimates, see Figure S3). This pattern of weights was not due to selfish and altruistic people’s results averaging out: 92 participants’ HDIs overlapped.

Second, Fairness on its own was not a meaningful predictor. When included in the model using Self and Other Disparities as predictors, the conditional influence of Fairness was still credibly null (*μ*_βFairness_ HDI included zero). However, Fairness improved the model’s predictive accuracy according to the LOOIC—although not the model’s fit to the observed data (Table 1). To clarify whether this predictive accuracy improvement was because Fairness captured meaningful interindividual differences in our sample rather than collinearity between parameters, we analyzed only those trials in which Fairness and Joint-cost minimization predicted different decisions (6,682 “unambiguous” trials).

**Figure 5.**
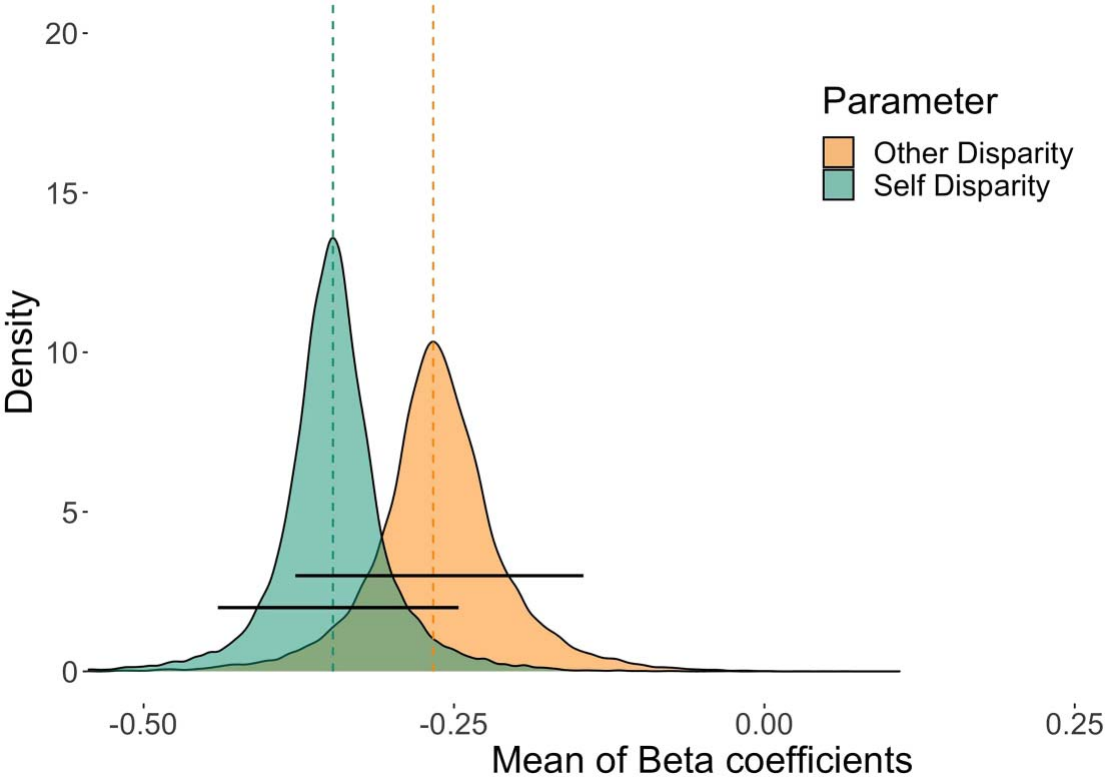
**Posterior probability distributions of the rescaled *μ*_β_ parameters in the Self + Other Disparity model estimated on the nonambiguous trials (Table 1, Model 3.2).** The dashed vertical lines indicate the modes *μ*_β_, the black horizontal lines represent the 95% HDIs (highest density intervals).

People made co-efficient choices in 76.6% (*SD* = 10.4) of these trials, almost exactly the same proportion as in the full sample. We reestimated the Self and Other Disparity and Fairness only models on this data set and found that the former predicted the participants’ behavior better (Table 1, Models 3.2 and 4.2). The estimates support the hypothesis that people made decisions that aimed to minimize both Self and Other costs (Figure 5; see Figure S4 for individual estimates). Overall, we found no clear effect of Fairness on decision making, and conclude that the Self and Other Disparities model provides the most accurate description of our findings.

### Learning and Tit-for-Tat Strategy

Were participants’ decision-making strategies stable over time? Did a partner’s previous co-efficient choices drive behavior as a tit-for-tat strategy? We addressed these questions by running additional models, extended with the factors Trial and Block (of five trials), and a variable coding whether the co-actor chose co-efficiently on their previous trial (PrevCoeff). We found that neither predictor improved the best model’s predictive accuracy or fit to the data (Table S6.1).

**Figure 6.**
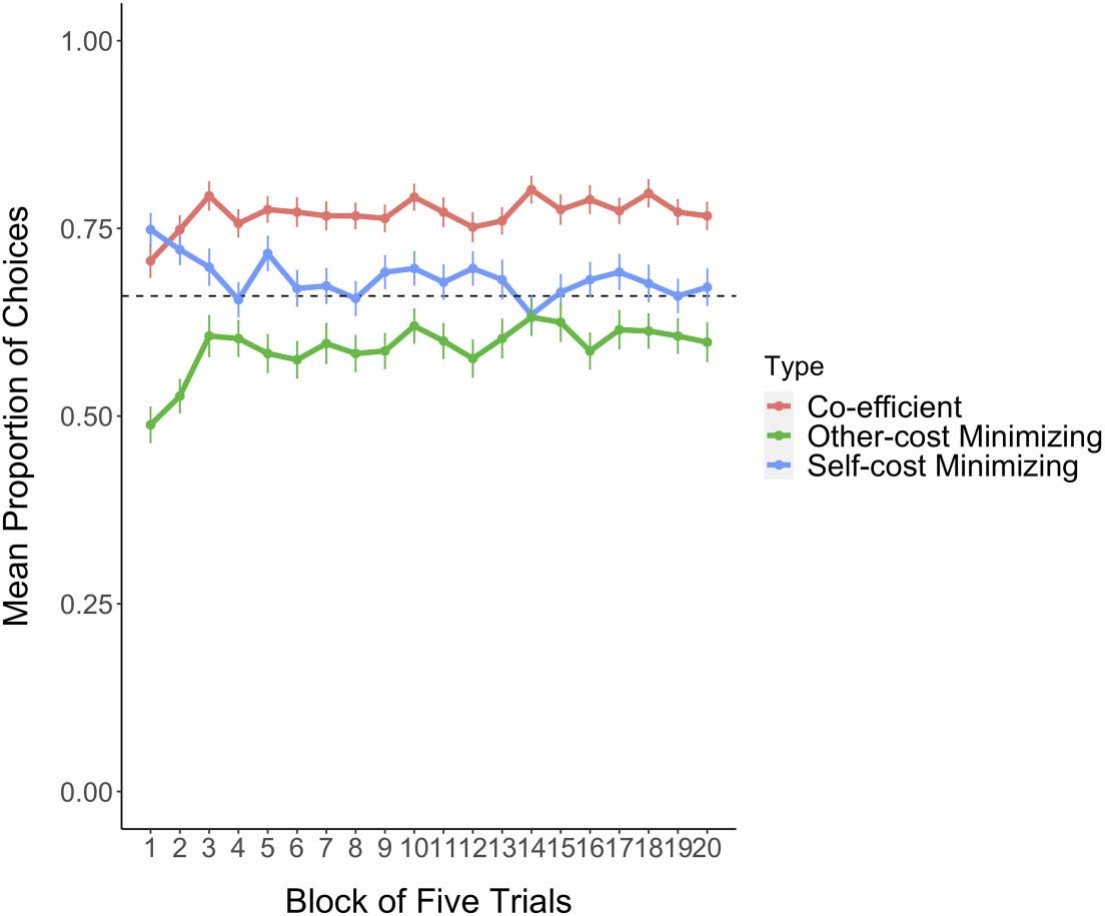
**Mean proportions of Joint-, Self-, and Other-cost minimizing object choices in five-trial blocks.** Each choice could be categorized in either or all of these categories due to overlaps between the predictions of each strategy in a trial. Error bars represent *SE*, the dashed horizontal line shows chance level.

Additionally, we reestimated all models on each participant’s first five decisions (Block 1) and found that in the first minute of a game, decisions were best described by the Self and Other Disparity model, although with a higher relative weight on Self costs (Self: .73, 95% HDI = [.47, 1.00], Other: .27, 95% HDI = [0, .53]; Table S6.1, Figure S5). These results together suggest that participants adopted a stable Joint-cost minimizing strategy following a brief phase of relative Self-cost minimization (Figure 6).

### Benefits of Co-efficient Decisions

To investigate whether co-efficient choices conferred any benefit to the dyad, we averaged the total trial durations for each dyad. When making co-efficient choices, dyads completed trials on average in *M*_Means_ = 4.55 s (*SD*_Means_ = 0.63 s), with an average standard deviation of *M*_*SD*_ = 0.98 s (*SD*_*SD*_ = 0.68 s). Trials with sub-efficient object choices numerically lasted longer for 55 out of 60 dyads, on average for *M*_Means_ = 4.78 s (*SD*_Means_ = 0.65 s), with a lower average standard deviation than in the case of co-efficient choices (*M*_*SD*_ = 0.83 s; *SD*_*SD*_ = 0.25 s). These results suggest a beneficial effect of co-efficient choices on task completion time. Statistical testing was not conducted due to the low number of sub-efficient choices.

## DISCUSSION

The current study explored the computations that underlie joint-cost minimizing decisions in planning joint actions. We tested the hypothesis that co-actors represent the collaborating dyad’s joint costs as a sum of the members’ individual costs and seek to minimize this value.

Participants made binary decisions between action plans with different associated movement costs in a joint object matching task. We modeled the parametric dependence of participants’ choices on the action cost disparities for the acting participant and those for her partner using mixed-effects logistic regression models estimated in the Bayesian framework. The cost disparities of the actor and co-actor were found to be similarly weighted, which suggests that the decision maker aimed to minimize the joint action cost (77% of choices were co-efficient).

We also tested an alternative hypothesis, according to which the minimization of unfairness in the distribution of individual action costs determines action decisions. Overall, we conclude that fairness did not influence action choices. Furthermore, we did not find conclusive evidence for participants following a tit-for-tat strategy along the lines of “If you choose co-efficiently, I will do so, too.” The results suggest that after briefly overweighting Self costs in the first few trials, the participants followed a Joint-cost minimizing strategy throughout the task.

Therefore, our experiments’ results provide support for co-efficiency maximization as a primary strategy of action planning in a joint task involving two contributions. This is consistent with the way Kleiman-Weiner and colleagues (2016) operationalized cooperative planning in their computational model, and confirms that, as long as the individual costs can be estimated on the same scale (i.e., as proportional to distance in our case), joint costs are calculated as the weighted sum of individual costs in joint action planning. Our findings suggest weights of ∼.56 on the decision maker’s own, and ∼.44 on her partner’s individual costs. The behavior we found is also qualitatively consistent with previous findings from a joint action task (Török et al., 2019) and economic games (Colman et al., 2008). Based on results from the co-representation literature (e.g., Schmitz et al., 2017), we speculate that in simultaneous tasks, too, people might account for joint costs.

It is possible that participants made choices based on hypothetical costs. However, we do not consider this a problem for our account. We argue that if participants had treated this as an abstract problem with more and less appropriate choices in a hypothetical mode (akin to a distance judgment task without movement), the fact that participants minimized movement distances for themselves and their partners suggests that the manipulation of even imaginary action costs influenced decision making. This would strengthen our account, not weaken it.

Investigating the factors that might modulate how actors’ individual action costs are weighed in decision making awaits future research. Relevant factors might include the explicit role distribution of co-actors, social hierarchies (Kleiman-Weiner et al., 2016), the relative competence of the co-actors at specific motor tasks, and uncertainty about the co-actors’ cost functions. Increasing the uncertainty about the partner’s action costs might make people downplay the importance of a co-actor’s individual costs in the computation of joint costs, or to ignore them altogether. Furthermore, more extreme costs or larger asymmetries between individuals might have similar effects on decision making: the former could push people toward self-interest (and so could social hierarchy), the latter could inspire a fairness-focused strategy instead. The effects of these factors should be explored to achieve a fuller understanding of the computations that people employ in cooperative action planning. As a first step toward this goal, the present study provides clear evidence for an additive cost computation that enables efficient coordination for a dyad in a sequential cooperative activity.

## ACKNOWLEDGMENTS

We thank Dávid Csu˝rös, Vanda Derzsi, and Fruzsina Kollányi for their assistance in data collection.

## FUNDING INFORMATION

NS, FP7 Ideas: European Research Council (https://dx.doi.org/10.13039/100011199), Award ID: 616072. GC, H2020 European Research Council (https://dx.doi.org/10.13039/100010663), Award ID: 742231.

## ROLE INFORMATION

GT: Conceptualization: Equal; Data curation: Equal; Methodology: Equal; Software: Equal; Writing – original draft: Lead; Writing – review & editing: Equal. OS: Conceptualization: Equal; Data curation: Equal; Methodology: Equal; Software: Equal; Writing – original draft: Equal; Writing – review & editing: Equal. NS: Conceptualization: Equal; Funding acquisition: Lead; Methodology: Equal; Supervision: Lead; Writing – original draft: Equal; Writing – review & editing: Equal. GC: Conceptualization: Equal; Funding acquisition: Lead; Methodology: Equal; Software: Equal; Supervision: Lead; Writing – original draft: Equal; Writing – review & editing: Equal.

## Notes

^1^ Despite its sequential nature, we consider this a joint action. For an action to qualify as joint action, a goal that is not individual but shared between co-actors should be present. In our task, the interdependence of the two individual actions to reach the goal of matching object pairs ensures that there is a joint goal.^2^ In 66% of the trials, the predictions of Self- and Joint-cost minimization overlapped, therefore we used that as chance level.^3^ For three comparisons to chance, α-levels were Bonferroni-corrected for repeated testing (α = .017). As a measure of effect size, we report rank-biserial correlations (Kerby, 2014).

## Supplemental Material

https://osf.io/r6mz3/?view_only=3f5fc782dac242adbe02bf3bc48158b0
