# Supplementary material for: Computing Joint Action Costs: Co-Actors Minimize the Aggregate Individual Costs in an Action Sequence
Source: Open Mind (Camb). 2021 Sep 10;5:100–12. doi: 10.1162/opmi_a_00045 (PMC8563062; doi:10.1162/opmi_a_00045)
Supplement: Supplementary file 1 [file opmi-05-100-s001.pdf]

Supplementary Material

S1. Methods – Additional Experiment-wise Information

S1.1. Participant exclusions

We excluded dyads (1) due to computing errors caused by equipment failure, which on occasion resulted in multiple disruptions during data collection; or (2) when the correlational structure of the experiment's parameters was not as intended, due to the design's stochastic nature; or (3) when participants chose the same object in every trial. Table S1.1 shows the total number of participants per experiment and the specific reasons for exclusion.

Table S1.1. Excluded participants

Experiment	Total number of participants	Number of excluded participants	Reasons for exclusion
1	48 (24 dyads)	8 (4 dyads)	equipment failure (3); unintended correlation in parameters (1)
2	66 (33 dyads)	26 (13 dyads)	equipment failure (13)<br><br>7 dyads were included in the final dataset who whose sessions were disrupted once, but successfully resumed (results from this sample were consistent with those form the rest of the group)
3	52 (26 dyads)	12 (6 dyad)	equipment failure (1), lost data (4), participant compliance (1)

S1.2. Design

The three experiments we conducted were different only in terms of the parameter pairs that were de-correlated from one another across trials. In Experiment 1, the individual Self and Other cost disparities were statistically independent from one another. In Experiment 2, Actor

1's individual costs were independent from the joint action costs; whereas in Experiment 3, the second actor's individual costs were independent from the joint costs. Here we describe the parameter sampling procedures applied in each experiment.

S1.2.1. Experiment 1. In Experiment 1, our primary aim was to investigate the independent contribution of Self Disparity and Other Disparity to the actors' decisions. In order to make this possible, we kept the distributions of these two factors uncorrelated across trials. Thus, to generate the locations of the target objects, we sampled Self Disparity and Other Disparity for each trial independently from the same uniform distribution (between -265 and 265 pixels)[1]. We then randomly selected the positions of all objects in such a way as to match these disparities and with the constraints that (1) the distance between the starting positions and the objects be between 120 and 385 pixels[2], (2) the angle between the line from an object to the start position and the edge of the screen be a minimum of 15°, (3) the angular separation between the paths from the starting positions to the objects be at least 45°, (4) and the absolute distance between the objects on both sides be at least 124 pixels.

The sampling process that generated object arrangements guaranteed that Self Disparity and Other Disparity were uncorrelated (Fig. S1a). However, as a direct consequence, Joint Disparity (the sum of the two individual disparities) had a triangular distribution and was positively correlated with both terms (Figs. S1b-c).

[1] The script is available on the OSF site of the project: https://osf.io/r6mz3/?view_only=3f5fc782dac242adbe02bf3bc48158b0
[2] On the screen, 100 pixels were equal to approximately 5.3 cm.

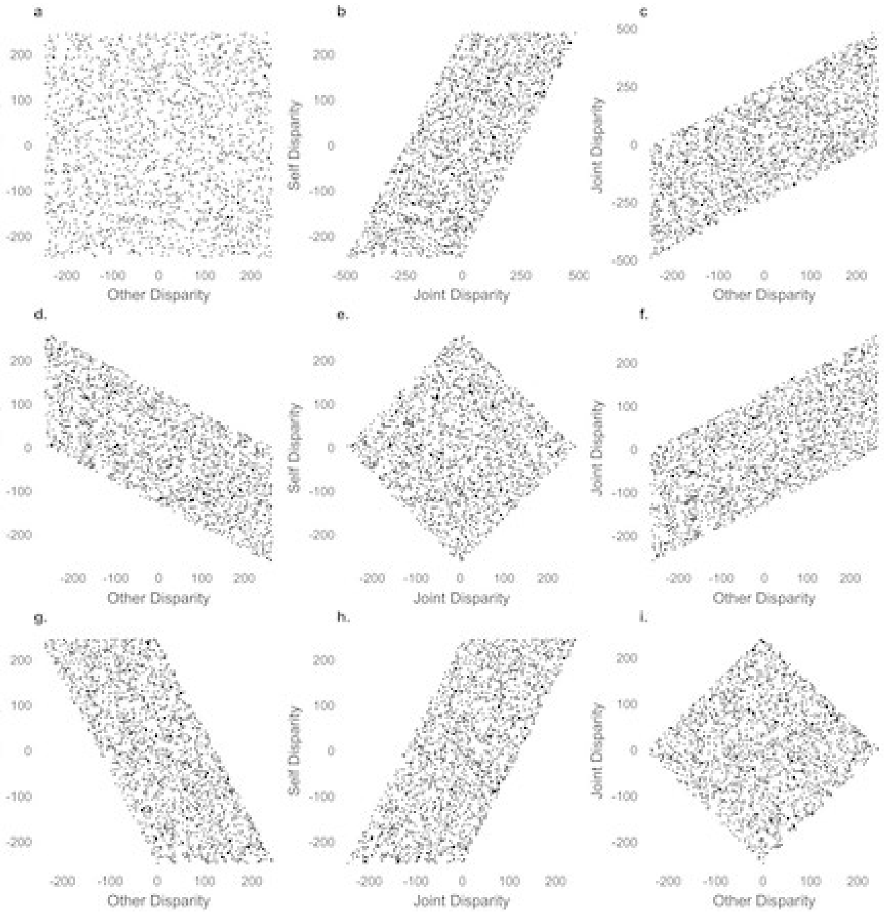

Fig. S1. Scatterplots of the joint distributions of cost disparities in (a-c) Experiment 1, collapsed across all trials of all dyads (20 dyads, 2000 trials). (a) Self and Other Disparities were uncorrelated. (b) Self and Joint Disparities, and (c) Joint and Other Disparities were positively correlated with each other. (d-f) In Experiment 2, (d) Self and Other Disparities were negatively correlated with each other, (e) Self and Joint Disparities were uncorrelated, and (f) Joint and Other Disparities were positively correlated with each other. (g-i) In Experiment 3, (g) Self and Other Disparities were negatively correlated, (h) Self and Joint Disparities were positively correlated with each other, and (i) Joint and Other Disparities were uncorrelated.

S.1.2.2. Experiments 2 and 3. Experiment 2 tested the hypothesis that action initiators (Actor 1) plan their movements to minimize the summed aggregate action costs of the dyad's action sequence (*Joint Cost Disparity*) rather than to minimize their own individual costs (*Self Cost Disparity*). Experiment 3 probed the effect of Joint Cost Disparity against the individual costs of Actor 2 (*Other Cost Disparity*).

In both additional experiments, we applied the task from Experiment 1, and generated the layouts with the target objects' locations in the same way as in Experiment 1, with some important changes. We first sampled the individual – Self in Experiment 2, and Other in Experiment 3 – Cost Disparities for each trial from a triangular distribution with mode = 0 and

limits provided by the maximum possible distance between an Actor's starting position and any target object (-265, 265 pixels). Then the parameters for Actor 2 (Other Disparity, Experiment 2) and Actor 1 (Self Disparity, Experiment 3), respectively, were drawn from a uniform distribution with limits set using the initially sampled Disparity parameter multiplied by -1.

Due to these sampling steps, the two actors' individual costs were negatively correlated with each other in both experiments (Fig. S1d, S1g), and the Joint Disparity defined by the two individual parameters' sum was independent from the Self Disparity (and positively correlated with Other Disparity, Figs. S2e-f) in Experiment 2, whereas it was independent from the Other Disparity in Experiment 3 (and positively correlated with Self Disparity, Figs. S1h-i). As in Experiment 1, every dyad in both experiments completed 200 trials (100 uniquely generated trials per participant) in a pseudo-random order.

S1.3. Description of the hierarchical model

We assumed that the trial-by-trial probability of choosing object A1 was Bernoulli distributed with parameter $\mu_{i|s,k}$, where *i* indexes the trial, *s* indexes the participant and *k* indexes the experiment that the participants participated in (see Trial level in Fig. S2.). The value of this parameter depended on a logistic function of the focal cost parameter(s) of the model weighted by the participant's β coefficient/s, $\beta_{Self,s,k}$, $\beta_{Other,s,k}$ or $\beta_{Fairness,s,k}$ (Subject level). The intercept was not estimated in the models, which is equivalent to assuming random decisions in the absence of any action cost disparities.

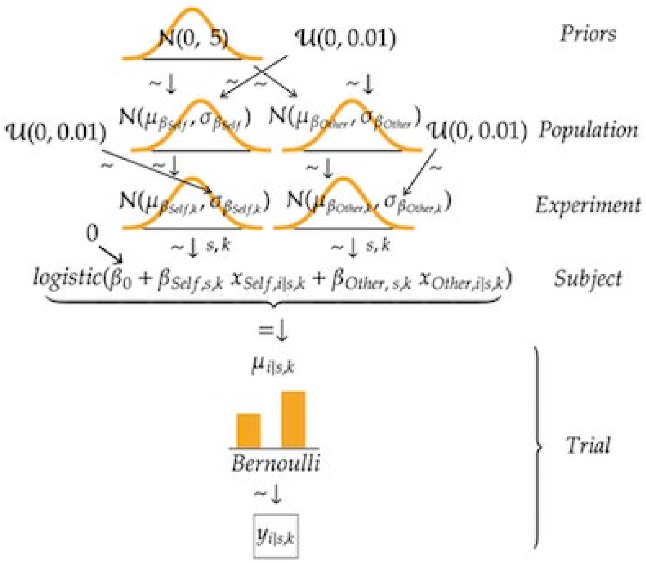

Fig. S2. A graphical schema of the hierarchical regression model, adapted from Kruschke (2015).

The individual β coefficients were assumed to be normally distributed at the Experiment level around means $\mu_{\beta Self,k}$, $\mu_{\beta Other,k}$, and $\mu_{\beta Fairness,k}$ with standard deviations $\sigma_{Other,k}$, $\sigma_{Self,k}$, and $\sigma_{Other,k}$, corresponding to the assumption that participants' individual weighing strategies are noisy versions of a shared group-level weighing pattern within an experiment. We included a Population level above the Experiment level with μ_β and σ_β values for each cost parameter's β coefficients. Each experiment's $\mu_{Self,k}$, $\mu_{Other,k}$, $\mu_{Fairness,k}$, $\sigma_{Self,k}$, $\sigma_{Other,k}$, and $\sigma_{Fairness,k}$ parameters were assumed to be sampled from the Population level, e.g. $\mu_{\beta Self,k} \sim \mathcal{N}(\mu_{\beta Self}, \sigma_{\beta Self})$ and $\sigma_{\beta Self,k} \sim \mathcal{U}(0.0, 0.01)$. The priors for the Population level were set by hyperparameters $\mu_{\beta Self} \sim \mathcal{N}(0, 5)$ and $\sigma_{\beta Self} \sim \mathcal{U}(0.0, 0.01)$ (similarly for the other disparity parameters), a distribution around a zero effect of cost disparity. The priors for the σ_β parameters (and for the $\sigma_{\beta,k}$, parameters one level below) were set to approximately match the ranges of posterior σ_β estimates of the initial

experiment-wise analyses[3] (Priors level). The same hyperpriors were used for all the predictors across all models.

S1.4. Technical information on the estimation process

We customized Bayesian data analysis scripts that are freely available online to accompany Kruschke (2015)[4]. Specifically, we adapted a multiple logistic regression model (Kruschke, 2015, p. 622) to predict a categorical dependent variable (object choice) in a hierarchical structure, which enabled the simultaneous estimation of individual, experiment-, and population-level β coefficient distributions.

All models were estimated using a Gibbs sampler in the runjags package (Denwood, 2016) in R (version 3.5.1). Three chains were initialized using fixed seeds of three random number generators for the reproducibility of results. At first, 1,000 adaptation steps and 10,000 burn-in steps were taken and discarded before reaching convergence between the three chains. We kept 29,000 subsequent iterations for analysis, by thinning out every second step. Chain convergence was checked using Gelman and Rubin's (1992) convergence diagnostic, the potential scale reduction factor (PSRF). In most of the models, this factor's value was close to 1, i.e., chain convergence was satisfactory, and the full range of posterior distributions were explored. Although increasing the chain size would have ensured that all models' PSRF values be around 1, we had to compromise by capping the chain length at 29,000 iterations due to finite computational resources (to enable the calculation of WAIC and LOOIC measures for

[3] N.B. Where comparison was possible, the experiment-level estimates did not qualitatively differ between the pooled analyses reported in the main text and in section S2 (Table S2.1), and the original, experiment-wise, analyses (reported in Table S3.1). The original hyperpriors used for each experiment were $\mu \sim (0, 2)$ and $\sigma \sim (0.0, 0.5)$. See section S.3 for details.

[4] The software and scripts were downloaded from https://sites.google.com/site/doingbayesiandataanalysis/software-installation .

model comparison, we had to estimate the log-likelihood at each trial, which placed considerable strain on our technical resources).

The data collected in the three experiments of the present study and the analysis scripts are available on the OSF site of the project:

https://osf.io/r6mz3/?view_only=3f5fc782dac242adbe02bf3bc48158b0

References

Denwood, M. J. (2016). runjags: An R Package Providing Interface Utilities, Model Templates, Parallel Computing Methods and Additional Distributions for MCMC Models in JAGS. *Journal of Statistical Software, 71(9)*, 1-25. doi:10.18637/jss.v071.i09

Gelman, A., & Rubin, D. B. (1992). Inference from iterative simulation using multiple sequences. *Statistical Science, 7*, 457-511.

Kruschke, J. (2015). *Doing Bayesian data analysis: A tutorial with R, JAGS, and Stan* (2nd ed.). Academic Press.

S2. Results – Additional Experiment-wise Information

We report the experiment-level parameter estimates for the eight logistic regression models reported in the main text. First, Table S2.1 summarizes these, together with the population-level estimates and measures of model fit; then follows a detailed description of the results of the five main models.

Table S2.1. Raw (pixel-based) parameter estimates and measures of predictive accuracy and model fit (WAIC – Watanabe-Akaike Information Criterion, LOOIC – Leave-one-out Information Criterion, AUC - Area Under the Curve) of the logistic regression models. Each row reports either population- or experiment-level estimates (indicated in the first column) for a given model.

Model	μ_β Mode	μ_β 95% HDI	σ_β Mode	σ_β 95% HDI	WAIC [SE]	LOOIC [SE]	AUC
**Model 1: Self Disparity**					13108.7 [97.3]	13109.1 [97.3]	0.735
Population-level	-0.008	-0.017, -0.0003	0.006	0.003, 0.010			
Experiment 1	-0.012	-0.015, -0.010	0.009	0.007, 0.010			
Experiment 2	-0.002	-0.004, 0.0003	0.006	0.005, 0.009			
Experiment 3	-0.011	-0.013, -0.009	0.006	0.005, 0.008			
**Model 2: Other Disparity**					14529.0 [82.0]	14529.2 [82.0]	0.628
Population-level	-0.003	-0.011, 0.005	0.006	0.003, 0.010			
Experiment 1	-0.007	-0.010, -0.005	0.007	0.005, 0.009			
Experiment 2	-0.006	-0.007, -0.004	0.005	0.004, 0.007			
Experiment 3	0.004	0.001, 0.006	0.006	0.005, 0.008			
**Model 3: Self + Other Disparity**					9436.5 [124.5]	9437.8 [124.6]	0.859
Population-level	Self: -0.019	-0.025, -0.014	0.001	4.14e-07, 0.008			

	Other: -0.015	-0.021, -0.010	0.001	2.88e-07, 0.008			
Experiment 1	Self: -0.019	-0.021, -0.016	0.009	0.007, 0.010			
	Other: -0.014	-0.017, -0.011	0.009	0.007, 0.010			
Experiment 2	Self: -0.019	-0.021, -0.016	0.006	0.005, 0.009			
	Other: -0.016	-0.019, -0.014	0.007	0.005, 0.009			
Experiment 3	Self: -0.021	-0.024, -0.018	0.007	0.006, 0.010			
	Other: -0.015	-0.018, -0.013	0.008	0.007, 0.010			
**Model 4: Fairness**					16153.8 [42.2]	16153.9 [42.2]	0.542
Population-level	-0.0001	-0.005, 0.005	0.002	0.001, 0.008			
Experiment 1	-0.001	-0.002, -0.0003	0.0001	1.03e-06, 0.001			
Experiment 2	0.002	0.001, 0.003	0.003	0.002, 0.004			
Experiment 3	-0.001	-0.001, 0.0004	0.001	3.14e-07, 0.002			
**Model 5: Self + Other Disparity + Fairness**					9406.3 [124.4]	9407.8 [124.5]	0.851
Population-level	Self: -0.020	-0.025, -0.014	0.001	3.81e-06, 0.008			
	Other: -0.015	-0.021, -0.010	0.001	8.24e-08, 0.008			
	Fairness: -0.002	-0.006, 0.004	0.001	8.98e-07, 0.008			
Experiment 1	Self: -0.019	-0.022, -0.016	0.008	0.007, 0.010			
	Other: -0.014	-0.017, -0.011	0.009	0.008, 0.010			
	Fairness: -0.002	-0.004, -0.001	0.002	3.28e-06, 0.004			
Experiment 2	Self: -0.020	-0.024, -0.013	0.006	0.004, 0.009			

	Other: -0.015	-0.022, -0.011	0.007	0.005, 0.009			
	Fairness: 0.001	-0.004, 0.005	0.0001	1.18e-07, 0.003			
Experiment 3	Self: -0.021	-0.024, -0.019	0.007	0.006, 0.010			
	Other: -0.016	-0.018, -0.013	0.009	0.007, 0.010			
	Fairness: -0.002	-0.003, -0.0003	0.003	0.001, 0.005			
**Model 3.2: Self + Other Disparity (unambiguous trials)**					5412.4 [93.4]	5414.6 [93.5]	0.855
Population-level	Self: -0.018	-0.023, -0.013	0.001	3.99e-06, 0.008			
	Other: -0.014	-0.020, -0.008	0.002	4.26e-07, 0.009			
Experiment 1	Self: -0.017	-0.020, -0.014	0.008	0.006, 0.010			
	Other: -0.012	-0.015, -0.009	0.009	0.007, 0.010			
Experiment 2	Self: -0.019	-0.021, -0.016	0.006	0.004, 0.009			
	Other: -0.016	-0.019, -0.014	0.006	0.005, 0.008			
Experiment 3	Self: -0.019	-0.022, -0.017	0.006	0.004, 0.009			
	Other: -0.014	-0.017, -0.011	0.008	0.006, 0.010			
**Model 3.3: Self + Other Disparity (Block 1)**					606.9 [27.0]	610.6 [27.2]	0.825
Population-level	Self: -0.016	-0.022, -0.010	0.001	5.05e-07, 0.008			
	Other: -0.006	-0.011, -2.16e-06	0.001	3.26e-07, 0.008			
Experiment 1	Self: -0.016	-0.021, -0.013	0.0003	9.35e-06, 0.008			
	Other: -0.005	-0.009, -0.002	0.009	0.003, 0.010			

Experiment 2	Self: -0.015	-0.020, -0.010	0.009	0.002, 0.010			
	Other: -0.007	-0.010, -0.004	0.003	0.0001, 0.007			
Experiment 3	Self: -0.016	-0.020, -0.012	0.008	0.002, 0.010			
	Other: -0.006	-0.009, -0.0003	0.003	4.64e-05, 0.009			
**Model 4.2: Fairness (unambiguous trials)**					7850.3 [74.9]	7850.9 [75.0]	0.748
Population-level	0.009	0.002, 0.017	0.004	0.003, 0.010			
Experiment 1	0.013	0.011, 0.015	0.003	0.001, 0.006			
Experiment 2	0.004	0.003, 0.006	0.004	0.003, 0.005			
Experiment 3	0.011	0.009, 0.013	0.004	0.001, 0.006			

S2.1. Models 1 and 2: Self Disparity, Other Disparity

Model 1: Self Disparity. In Experiments 1 and 3, Actor 1's individual cost disparities exerted non-zero effects on the probability of their choosing object A1, whereas in Experiment 2, this effect was not different from zero. In Experiments 1 and 3, the modes of the posterior distributions of $\mu_{\beta\text{Self},1}$ and $\mu_{\beta\text{Self},3}$, the parameters for the experiment-level coefficient for the cost disparity, were -0.235 (95% HDI: [-0.291, -0.186]), and -0.204 (95% HDI: [-0.244, -0.164]), respectively. For every onscreen centimeter increase in Self Disparity, a 20.9% (Experiment 1) and a 18.4% (Experiment 3) decrease in the odds of an object A1 choice was expected. In Experiment 2, the estimated 95% HDI of the posterior distribution of the $\mu_{\beta\text{Self},2}$ parameter included zero (Mode $\mu_{\beta\text{Self},2}$ = -0.038, 95% HDI: [-0.078, 0.006]). The modal decrease in the odds of picking object A1 over B1 with a one cm increase in Self Disparity was 3.7%.

Model 2: Other Disparity. The estimation of the experiment-wise $\mu_{\beta\text{Other},k}$ parameter's posteriors for the Other Disparity model found that Actor 2's cost disparity had negative, non-zero effects on the odds of object A1 choices in Experiments 1 and 2, whereas the estimates in Experiment 3 were distributed above zero.

In Experiments 1 and 2, the modes of the $\mu_{\beta\text{Other},1}$ and $\mu_{\beta\text{Other},2}$ parameter estimates were -0.137 (Experiment 1, 95% HDI: [-0.179, -0.097]) and -0.105 (Experiment 2, 95% HDI: [-0.137, -0.071]). This indicated that the expected decreases in the odds of an object A1 choice over a B1 choice, when Other Disparity increased by one centimeter, were 12.8% (Experiment 1) and 10.0% (Experiment 2). In Experiment 3, however, the posterior distribution of the Other Disparity's estimated $\mu_{\beta\text{Other},3}$ parameter was fully above zero: with Mode $\mu_{\beta\text{Other},3}$ = 0.066 (95% HDI: [0.026, 0.105]). This means that contrary to our predictions, increasing Other Disparity by one cm resulted in an expected increase of 6.8% in the odds of the participant picking A1 over B1.

To summarize, we found that in the case of the two single-predictor models, in 2 out of 3 experiments – when each of them was correlated with Joint Disparity – the disparities influenced decisions in the expected negative direction. The results of the estimations suggest that when each cost disparity parameter was de-correlated from the Joint Disparity of action sequences – i.e., Self Disparity in Experiment 2, and Other Disparity in Experiment 3 –, their effects were not as expected. Self Disparity by itself did not have an effect on choices (the 95% HDI included zero), whereas Other Disparity had an effect in the opposite direction than expected: when Other Disparity increased, the odds of an A1 choice also increased. This could possibly be due to an effect of self-cost minimization, because Other Disparity was negatively correlated with Self Disparity in Experiment 3.

S2.2. Model 3: Self and Other Disparities

In all three experiments, the experiment-level means ($\mu_{\beta Self,k}$ and $\mu_{\beta Other,k}$) of the β_{Self} and β_{Other} coefficients for both disparities in Model 3 were distributed below zero. In Experiment 1, the mode of the $\mu_{\beta Self,1}$ parameter's posterior distribution was -0.355 (95% HDI: [-0.405, -0.306]), and the mode of the $\mu_{\beta Other,1}$ posterior was -0.265 (95% HDI: [-0.316, -0.207]). In Experiment 2, the two modes were similar in magnitude (Mode $\mu_{\beta Self,2}$ = -0.354, 95% HDI: [-0.394, -0.307]; Mode $\mu_{\beta Other,2}$ = -0.307, 95% HDI: [-0.356, -0.268]), as were the estimates in Experiment 3, although to a lesser degree (Mode $\mu_{\beta Self,3}$ = -0.389, 95% HDI: [-0.445, -0.341]; Mode $\mu_{\beta Other,3}$ = -0.290, 95% HDI: [-0.343, -0.244).

Increasing Self and Other disparities (reported in this order) by a centimeter was expected to lead to a 29.9% and 23.3% decrease in Experiment 1, a 29.8% and 26.4% decrease in Experiment 2, and a 32.2% and 25.2% decrease in Experiment 3 in the odds of picking object A1 over B1. The 95% HDIs of the coefficients of the two cost disparities overlapped with one another in all three experiments, suggesting that there were no substantial differences between

the magnitudes of the effects of the Self and Other disparities on decision-making. The relative average weights on Self and Other Disparity in the joint utility according to this combination model were .57 (95% HDI: [.49, .65]) and .43 (95% HDI: [.33, .51]), respectively, in Experiment 1; .54 (Self Disparity, 95% HDI: [.46 .60]) and .46 (Other Disparity, 95% HDI: [.41, .54]) in Experiment 2; and .57 (95% HDI: [.50, .66]) and .43 (95% HDI: [.36, .51] in Experiment 3.

S2.3. Models 4 & 5: "Minimizing unfairness"

Model 4: Fairness only. The experiment-level estimates for the Fairness only model differed between the three experiments. In Experiment 1, we found a small non-zero effect of Fairness in the predicted direction (Mode $\mu_{\beta\text{Fairness},1}$ = -0.024, 95% HDI: [-0.035, -0.006]). This suggests that with a one cm increase in the asymmetry in cost distribution between the two co-actors, a 2.4% decrease in the odds of an object A1 choice over B1 was expected.

In Experiment 2, we found a small effect in the opposite direction: the 95% HDI of the posterior distribution of the $\mu_{\beta\text{Fairness},2}$ estimates did not include zero, with a mode of 0.032 (95% HDI: [0.011, 0.052]). This means that a one cm increase in cost distribution asymmetry related to object A1 resulted in a 3.2% odds *increase* of picking A1 object over B1. Finally, in Experiment 3, the 95% HDI of the posterior distribution of the $\mu_{\beta\text{Fairness},3}$ estimates included zero ([-0.023, 0.008]), the most credible β coefficient was Mode $\mu_{\beta\text{Fairness},3}$ = -0.009. These results suggest that overall, Fairness did not influence the probability of Actor 1 picking object A1 in a consistent manner across the experiments.

Model 5: Self, Other Disparity and Fairness. In the other combination model, we found similar patterns of results across experiments. In Experiment 1, the estimated posterior distributions of the Self and Other Disparity parameters' $\mu_{\beta\text{Self},k}$ and $\mu_{\beta\text{Other},k}$ values were both entirely below zero and the 95% HDIs of the two distributions overlapped with each other (Self

Mode $\mu_{\beta Self,1}$ = -0.360, 95% HDI: [-0.411, -0.308]; Other Mode $\mu_{\beta Other,1}$ = -0.266, 95% HDI: [-0.316, -0.207]). In addition, we found a small non-zero effect of the Fairness parameter in the predicted negative direction (Mode $\mu_{\beta Fairness,1}$ = -0.042, 95% HDI: [-0.071, -0.018]). The expected odds decreases of an object A1 choice when each parameter was increased by one cm were 30.2% (Self Disparity), 23.3% (Other Disparity) and 4.1% (Fairness).

In Experiment 2, we found an even larger overlap between the effect sizes of Self and Other Disparity than in Experiment 1, suggested by a considerable overlap between the two 95% HDIs of the estimated posteriors (Self Mode $\mu_{\beta Self,2}$ = -0.371, 95% HDI: [-0.446, -0.254]; Other Mode $\mu_{\beta Other,2}$ = -0.288, 95% HDI: [-0.408, -0.216]). However, the effect of Fairness was not different from zero, suggested by the inclusion of 0 in the 95% HDI of the posterior distribution (Mode $\mu_{\beta Fairness,2}$ = -0.025, 95% HDI: [-0.081, 0.101]). By a one cm decrease in the Self and Other cost disparities, the odds of an A1 choice over the alternative decreased by 30.1% and 25.0%, respectively.

Finally, in Experiment 3, we found (similarly to Model 3) a small difference between the boundaries of the 95% HDIs of the posteriors of the μ_β coefficients on the Self and Other Disparities (Self Mode $\mu_{\beta Self,3}$ = -0.401, 95% HDI: [-0.454, -0.350]; Other Mode $\mu_{\beta Other,3}$ = -0.294, 95% HDI: [-0.347, -0.244]). This reflects the participants' tendency to minimize Self Disparities to a slightly larger extent than Other Disparities in Experiment 3 – which was also reflected in the positive coefficients on the Other Disparity in Model 2 (Other Disparity only model). Nevertheless, the results were similar to those in Experiment 1, in that the estimated beta weights on Self and Other Disparity were both much smaller than zero, in combination with a very small Fairness effect (Mode $\mu_{\beta Fairness,3}$ = -0.032, 95% HDI: [-0.057, -0.006]). Increasing each parameter by one cm resulted in expected decreases in the odds of an A1 choice by 33.0% (Self Disparity), 25.5% (Other Disparity), and 3.1% (Fairness).

Overall, the estimated weights on each parameter in the joint utility function, according to the combination model, were similar to one another across the three experiments. In Experiment 1, the weight on Self Disparity was .54 (95% HDI: [.46, .62]), on Other Disparity: .40 (95% HDI: [.31, .47]), and on Fairness, it was .06 (95% HDI: [.03, .11]). The values of these weights estimated based on Experiment 2's data were .59 (95% HDI: [.40, .70]) on Self Disparity, .45 (95% HDI: [.34, .64]) on Other Disparity, and .04 (95% HDI: [-.16, .13]) on Fairness. Finally, we found that in Experiment 3, the weights were .55 (Self Disparity, 95% HDI: [.48, .62]), .40 (Other Disparity, 95% HDI: [.34, .48]), and .04 (Fairness, 95% HDI: [.01, .08]).

S3. Additional Experiment-wise Information: Separate analyses

We report in Table S3.1. the results of the original parameter estimations that we conducted on each experiment's data before pooling them together for the unified analyses. Since the designs of the three experiments differed in which pairs of cost disparities were de-correlated from one another, the models estimated also differed in the parameter combinations we used as predictors. Multiple-predictor models only included de-correlated parameter pairs; and in Experiments 2 and 3, we estimated only those single-predictor models of which the predictors were independent from Joint Disparity (i.e., in Experiment 2, we estimated only the Self Disparity model, in Experiment 3, only the Other Disparity model). In Experiment 1, although both individual cost disparity parameters were correlated with Joint Disparity, they were each tested as predictors in single-predictor models (Self Disparity only, Other Disparity only) to measure their predictive power against the combination model Self + Other Disparity.

S3.1. Description of the experiment-wise hierarchical models

The experiment-wise models were identical in structure to the described pooled data model, except for the removal of the experiment level.

We set the uninformed priors for this group-level distribution by vague hyperparameters ($\mu \sim (0, 2)$, $\sigma \sim (0.0, 0.5)$), a wide distribution around a zero effect of cost disparity. The same uninformed hyperprior was used for all cost disparities, expressing our prior expectation that participants would weigh the minimization of all costs equally (Priors level).

Table S3.1. Raw (pixel-based) parameter estimates and model fit measures (DIC – Deviance Information Criterion, AUC – Area Under the Curve) of the original experiment-wise logistic regression models. The best fitting models' estimates for each experiment are set in bold.

Model	μ_β Mode	μ_β 95% HDI	σ_β Mode	σ_β 95% HDI	DIC	AUC
**Experiment 1**						
1: Self Disparity	-0.013	-0.016, -0.010	0.009	0.007, 0.012	3810	0.817
2: Other Disparity	-0.008	-0.010, -0.005	0.006	0.005, 0.009	4575	0.723
**3: Self + Other Disparities**	**Self: -0.019**	**-0.022, -0.016**	**0.009**	**0.006, 0.012**	**2467**	**0.898**
	**Other: -0.013**	**-0.016, -0.010**	**0.009**	**0.007, 0.012**		
4: Fairness	-0.001	-0.002, -0.0004	0.0002	3.57e-08, 0.002	5539	0.526
5: Joint Disparity + Fairness	Joint: -0.013	-0.014, -0.011	0.004	0.003, 0.005	3205	0.893
	Fairness: -0.002	-0.004, -0.001	0.002	3.92e-07, 0.003		
**Experiment 2**						
1: Self Disparity	-0.002	-0.004, 0.001	0.006	0.005, 0.008	5231	0.542
2: Joint Disparity	-0.016	-0.018, -0.014	0.006	0.005, 0.009	4023	0.822
**3: Joint + Self Disparities**	**Joint: -0.017**	**-0.020, -0.014**	**0.007**	**0.005, 0.010**	**3637**	**0.824**
	**Self: -0.002**	**-0.004, 0.001**	**0.008**	**0.006, 0.011**		
**Experiment 3**						
1: Other Disparity	0.004	0.002, 0.006	0.006	0.005, 0.008	5202	0.593
2: Joint Disparity	-0.019	-0.022, -0.016	0.007	0.005, 0.010	3821	0.843
**3: Joint + Other Disparities**	**Joint: -0.022**	**-0.026, -0.019**	**0.009**	**0.006, 0.013**	**3317**	**0.857**
	**Other: 0.006**	**0.003, 0.009**	**0.009**	**0.007, 0.012**		

S4. Correlations between Perspective-Taking, Empathy, Liking a Co-actor and Behavior Data

It is possible that general abilities of perspective-taking and empathic concern in social interactions may prove useful in the computation of collective action costs in cooperative contexts. We report the results of exploratory correlational analyses (conducted on the pooled data) of the potential relationships between how much participants prioritized joint-cost minimization and their perspective-taking abilities and degree of empathy towards other people, as well as how much they liked their co-actors.

Following the object matching task and before being debriefed about the experiment, the participants responded to a short custom questionnaire on their perceived purpose of the study and how much they liked their partner (“How much did you like your co-player?”). Ratings of Liking the partner were obtained using a 7-point Likert scale (1 – Not at all, 7 – Very much). Participants also completed the Perspective-Taking and Empathic Concern scales from the Davis Interpersonal Reactivity Index (Davis, 1980) as measures of perspective-taking and trait empathy. The maximum score on both scales was 28.

To operationalize the weight that participants placed on minimizing the joint costs of an action sequence, we used each participant’s proportion of co-efficient choices out of the 100 trials they completed as the decision-making Actor 1 (“co-efficiency ratio”). The higher the value of this measure, the bigger the weight a participant placed on minimizing the joint costs of an action sequence.

The average co-efficiency ratio was M = .77 (Range: .55 - .91, SD = .07). We found no statistically significant correlation between this measure and the Liking scores (Mdn = 6, interquartile range, IQR = 1, Spearman’s $\rho = .080$, $p = .387$). Likewise, we found no relationship

between the co-efficiency ratio and either Perspective-Taking (Mdn = 19, IQR = 5, ρ = -.038, p = .681) or Empathic Concern (Mdn = 20, IQR = 7, ρ = -.064, p = .486). These results suggest that in the present task, joint-cost minimizing behavior was unrelated to the participants' perspective-taking or empathic abilities and to how sympathetic they found their co-actor.

References

Davis, M. H. (1980). A multidimensional approach to individual differences in empathy. *JSAS Catalog of Selected Documents in Psychology, 10*, 85.

S5. Figures showing participant-wise estimates for the best-fitting models

The figures below present the individual-level parameter estimates according to the best-fitting model. Fig. S3. shows the results of the analysis on the entire dataset, Fig. S4. on the data subset where the predictions of fairness and co-efficiency were dissociated, and Fig. S5. on the first Block of 5 trials.

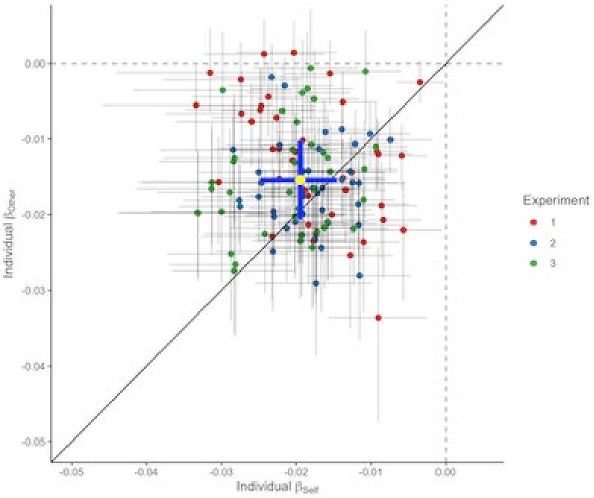

Fig. S3. Individual posterior estimates of the raw $\mu_{\beta Self,k}$ and $\mu_{\beta Other,k}$ parameters in the Self and Other Disparity model (Model 3) run on the whole dataset of the three experiments. Each individual's posterior modes are shown with the 95% HDIs. The dashed horizontal and vertical lines indicate zero.

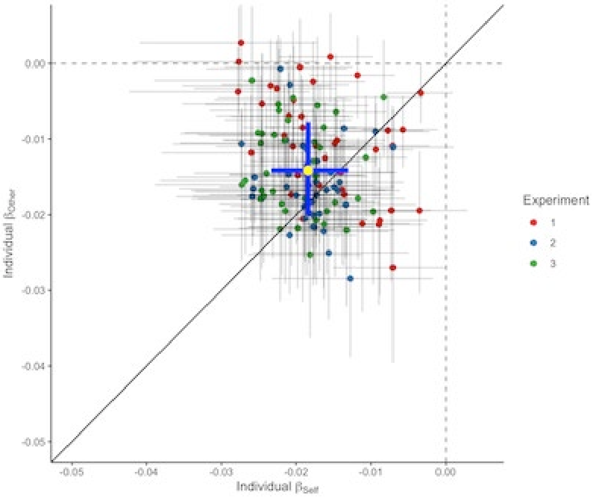

Fig. S4. Individual posterior estimates of the raw $\mu_{\beta Self,k}$ and $\mu_{\beta Other,k}$ parameters in the Self and Other Disparity model (Model 3.2) run on the unambiguous trials only where the predictions of Fairness and Joint-cost minimization diverged. Each individual's posterior modes are shown with the 95% HDIs. The dashed horizontal and vertical lines indicate zero.

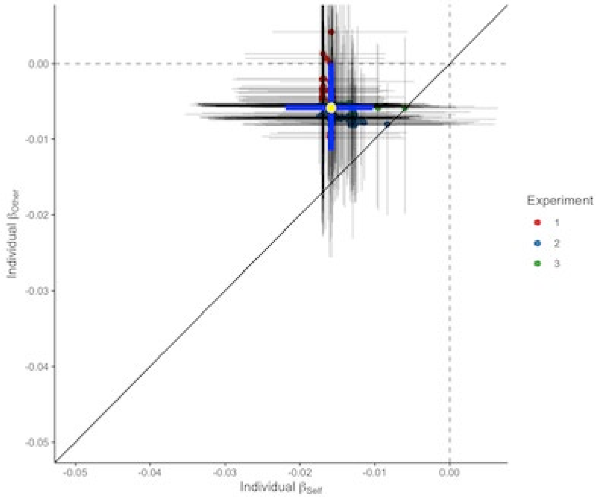

Fig. S5. Individual posterior estimates of the raw $\mu_{\beta Self,k}$ and $\mu_{\beta Other,k}$ parameters in the Self and Other Disparity model (Model 3.3) run on the first 5 trials (Block 1) of each participant playing as Actor 1. Each individual's posterior modes are shown with the 95% HDIs. The dashed horizontal and vertical lines indicate zero.

S6. Additional models examining the effect of learning and reciprocity of co-efficient decisions on strategy use

Table S6.1. Measures of predictive accuracy and model fit (WAIC – Watanabe-Akaike Information Criterion, LOOIC – Leave-one-out Information Criterion, AUC - Area Under the Curve) of all of the logistic regression models mentioned in the main text. We include the 5 main models and the extended models addressing questions of learning and tit-for-tat decision-making.

**Object choice (A1) predictors**	**Whole Session**			**First Block (5 trials)**		
	WAIC [SE]	LOOIC [SE]	AUC	WAIC [SE]	LOOIC [SE]	AUC
(1) Self Cost	13108.7 [97.3]	13109.1 [97.3]	0.735	642.7 [24.8]	644.8 [25.1]	0.797
1. PrevCoeff + Self	12211.0 [109.8]	12211.9 [109.8]	0.776	636.2 [25.6]	640.3 [25.9]	0.803
Self * Trial	13711.5 [94.4]	13712.7 [94.4]	0.714	654.2 [26.1]	670.4 [27.9]	0.788
Self * Block	13653.1 [95.0]	13654.2 [95.1]	0.716	N/A	N/A	N/A
(2) Other Cost	14529.0 [82.0]	14529.2 [82.0]	0.628	801.6 [11.9]	803.8 [12.0]	0.513
2. PrevCoeff + Other	13416.9 [100.6]	13417.4 [100.6]	0.717	768.2 [16.0]	773.8 [16.2]	0.622
Other * Trial	14743.2 [80.8]	14744.0 [80.9]	0.627	797.5 [12.7]	804.0 [13.2]	0.513
Other * Block	14712.3 [81.2]	14713.0 [81.2]	0.627	N/A	N/A	N/A
(3) Self Cost + Other Cost	9436.5 [124.5]	9437.8 [124.6]	**0.859**	606.9 [27.0]	**610.6 [27.2]**	0.825
3. PrevCoeff + Self + Other	9443.3 [124.6]	9444.7 [124.6]	0.859	610.2 [27.2]	616.4 [27.5]	0.826
Self * Trial + Other * Trial	10220.0 [128.7]	10224.6 [128.9]	0.843	615.1 [28.8]	641.7 [31.2]	0.813

(Self + Other) * Trial	11864.1 [112.8]	11864.9 [112.9]	0.839	701.5 [21.5]	711.5 [22.4]	0.754
Self * Block + Other * Block	10119.0 [129.1]	10123.8 [129.4]	0.844	N/A	N/A	N/A
(Self + Other) * Block	11788.2 [113.6]	11789.0 [113.7]	0.840	N/A	N/A	N/A
(4) Fairness	16153.8 [42.2]	16153.9 [42.2]	0.542	830.8 [5.8]	832.6 [5.9]	0.521
4. PrevCoeff + Fairness	14627.3 [85.1]	14627.6 [85.1]	0.699	796.7 [13.0]	801.5 [13.2]	0.625
Fairness * Trial	16165.6 [42.9]	16165.9 [42.9]	0.543	831.5 [6.9]	836.1 [7.2]	0.521
Fairness * Block	16159.7 [43.0]	16159.9 [43.0]	0.544	N/A	N/A	N/A
(5) Self Cost + Other Cost + Fairness	**9406.3 [124.4]**	**9407.8 [124.5]**	0.851	609.0 [27.1]	614.7 [27.5]	0.818
5. PrevCoeff + Self + Other + Fairness	9413.3 [124.6]	9415.1 [124.7]	0.851	611.5 [27.3]	619.4 [27.8]	0.816
Self * Trial + Other * Trial + Fairness * Trial	10182.9 [128.9]	10189.2 [129.2]	0.836	606.1 [28.6]	645.3 [31.7]	0.804
(Self + Other + Fairness) * Trial	13763.6 [92.2]	13764.3 [92.3]	0.710	734.4 [19.5]	743.9 [20.4]	0.719
Self * Block + Other * Block + Fairness * Block	10080.4 [129.3]	10086.8 [129.6]	0.834	N/A	N/A	N/A
(Self + Other + Fairness) * Block	13721.2 [92.9]	13721.9 [92.9]	0.711	N/A	N/A	N/A
(6) PrevCoeff	15050.6 [76.7]	15050.7 [76.7]	0.703	798.7 [11.6]	800.9 [11.7]	0.619
